# Should we modulate the neonatal microbiome and what should be the goal?

**DOI:** 10.1186/s40168-022-01281-4

**Published:** 2022-05-10

**Authors:** Niels van Best, Maria Gloria Dominguez-Bello, Mathias W. Hornef, Eldin Jašarević, Katri Korpela, Trevor D. Lawley

**Affiliations:** 1 Institute of Medical Microbiology, RWTH University Hospital Aachen, Aachen, Germany; 2 Department of Medical Microbiology, School of Nutrition and Translational Research in Metabolism (NUTRIM), Maastricht University, Maastricht, The Netherlands; 3 Departments of Biochemistry and Microbiology and of Anthropology, and Institute for Food, Nutrition and Health, Rutgers University, New Brunswick, NJ USA; 4 Department of Obstetrics, Gynecology and Reproductive Sciences, Magee-Womens Research Institute, University of Pittsburgh School of Medicine, Pittsburgh, PA USA; 5 Human Microbiome Research Program, Faculty of Medicine, University of Helsinki, Helsinki, Finland; 6 Wellcome Sanger Institute and Microbiotica, Cambridge, UK

Neonatal microbiome initialization and maturation have been a major focus of recent microbiome research. Perinatal influence factors, in particular the birth type by vaginal birth or Cesarean section, have been identified as a critical determinant of early-life microbiota composition and development. Different therapeutic approaches have been proposed and tested for neonatal microbiome restoration in C-section infants, which have received widespread scientific, clinical and public attention. To provide an overview and broader context of the current state of the scientific discourse, *Microbiome* asked several experts in the field to present their perspectives on neonatal microbiota establishment and maturation, the impact of C-sections, and the potential for early-life microbiome restoration. In an accompanying editorial, we also outline key questions and knowledge gaps around this topic [1].

## A neonatal microbiome of opportunity?

### Mathias W. Hornef and Niels van Best

After birth more than during any later time in life, the microbiome undergoes dramatic changes with rapidly rising bacterial density, major initial compositional fluctuations, the stepwise emergence of organ- and site-specific communities and a steady increase in bacterial diversity. The observed rapid rise in bacterial density at some anatomical sites is stunning and challenges our textbook knowledge on tissue homeostasis in the presence of microbial immune stimuli [2]. It may help to explain differences in the type and degree of the neonate’s innate immune response to infection [3]. It may also contribute to the etiology of necrotizing enterocolitis (NEC), a dysregulated proinflammatory response of the immature gut tissue of preterm neonates, illustrating the tightly intertwined relationship between tissue and cell development and host-microbial interaction.

The compositional fluctuations during the postnatal period most likely reflect the influence of endogenous or exogenous mechanisms on early bacterial colonizers, so-called pioneer bacteria [4–6]. Beside host-mediated selection, priority effects also appear to contribute to the early-life assembly of a stable gut microbial ecosystem [7–9]. The influence of specific bacterial species on the overall microbiome composition may therefore depend on the order and timing in which they arrive. Clearly, the mother represents a major source for the establishment of the microbiome consistent with the idea of the transgenerational transmission of an evolutionary optimized and beneficial assembly of microorganisms. Despite early reports on the role of the mother’s vaginal bacteria, the infant gut microbiota originates from the mother’s fecal microbiota most likely transmitted by the intense contact during the birth process [10, 11]. This early transmission event during vaginal delivery appears to be of key importance. A number of studies have shown that birth by C-section is associated with an increased colonization rate by opportunistic pathogens [12] as well as several immune-mediated diseases including inflammatory bowel disease, asthma, and juvenile arthritis [13, 14]. In addition, children delivered by primary c-section exhibit a higher risk of developing food allergy in later life [15]. This effect is independent of the perioperative antibiotic prophylaxis and thus most likely the result of an impaired bacterial transmission and altered immune imprinting [14–16]. Other factors such as diet (i.e. breastfeeding vs. formula feeding), siblings, pets and the natural environment also contribute in shaping the infant's microbiota and immune system but the identification of their individual contribution is challenging due to the presence of many confounders.

Another finding highlights the non-redundant role of the birth process as an early transmission event. In the adult host, different anatomical locations such as skin, gut or oral cavity are colonized by highly organ- and even site-specific communities. However, this anatomical specialization is not detected directly after birth but emerges only with time [5, 17]. This finding suggests the existence of organ-specific selective mechanisms, which remain largely uncharacterized. For example, our group showed that the microbiome composition was highly similar between the colon and small intestine early after birth but diverged into a site-specific microbiota after weaning. This anatomical specialization was paralleled by hepatic secretion of bile acids into the small intestinal lumen, a potential driver of microbiota selection [5]. Other potential drivers of the early small intestinal microbiome might represent the age-dependent switch in the spectrum of antimicrobial peptides or the Toll-like receptor (TLR)5-mediated suppression of colonization by flagellated bacteria [6, 18].

The initially reduced bacterial diversity opens ecological niches both for commensal and pathogenic bacteria and thereby explains the enhanced susceptibility of neonates to infection [19]. Most likely, it also explains why probiotic bacteria in early life exhibit at least some degree of colonization and exert much more pronounced effects than in adults [20]. The prolonged persistence of certain probiotic strains, especially bifidobacteria, exerts beneficial effects in neonates by increasing the colonization resistance and promoting a more stable microbial composition that protects against pathobiont-induced diseases such as NEC. In addition, the initially reduced bacterial diversity may render the neonate’s microbiota particularly susceptible to exogenous factors such as early life antibiotics and diet [21, 22].

The bacterial composition during early life appears to be of particular importance since specific microbial signals and metabolic factors influence the immune priming in a non-redundant fashion. Immune priming in turn determines the life-long susceptibility to immune-mediated and metabolic diseases [23–25]. Also host factors orchestrate the exposure of immune cells to luminal microbial stimuli and antigens. For instance, the decreasing epidermal growth factor (EGF) levels in breast milk around weaning foster mucosal translocation of microbial stimuli during the so-called ‘weaning reaction’ and induce enhanced expression of interferon (IFN)-ɣ and tumor necrosis factor (TNF)-ɑ and the generation of specific regulatory T cells [26]. This induction of specific regulatory T cells during the weaning reaction protects from inflammatory diseases in adults and represents an intriguing example of the combined influence of the microbiota, diet, and ontogeny in priming of the immune system and lifelong gut homeostasis.

Given the accessibility and functional importance, it is tempting to manipulate the early microbiota, i.e. by the administration of selected probiotics, prebiotics and postbiotics (bacterial metabolites) in order to improve the health outcome [20, 27, 28]. In addition, some efforts have been made in which neonates born by C-section were directly exposed to the maternal fecal microbiota upon delivery via fecal microbiota transplantation (FMT) to foster the maternal-neonatal microbiome transfer. However, our mechanistic understanding of the host regulatory effects, bacterial interactions, metabolic factors and influence of the microbiome on the neonate host are still incomplete. More mechanistic studies are needed to identify microbial or metabolic targets, establish interventional strategies, characterize possible adverse effects and define the patient groups that benefit most from safe and effective targeted interventions.

## Influence of the prenatal maternal microbiome on neonatal health?

### Eldin Jašarević

For more than a century, epidemiological studies demonstrated that maternal lifetime experiences, namely psychosocial stress, malnutrition, and infectious burden, predict poorer long-term outcomes across a variety of domains [29]. Offspring outcomes associated with these maternal exposures include higher risk for type 2 diabetes, obesity, cardiovascular disease, cancers, as well as neurodevelopmental and neuropsychiatric disorders [30, 31]. Disruption to the assembly and development of the neonatal microbiome is associated with similar negative health outcomes, catalyzing a rich body of literature examining the links between maternal lifetime exposures, the maternal and offspring microbiome, and lasting health trajectories [32–45].

A conceptual framework emerging from these studies suggests that environmental exposure during pregnancy alters maternal microbiota and these communities are subsequently transferred to offspring, influencing susceptibility to disease. One challenge in establishing causality between maternal exposures, microbiota, and offspring phenotype is that these environmental perturbations exert disruptive effects on both mother and developing fetus. In other words, offspring develop in a prenatal environment that is shaped by lifetime exposures to environmental conditions, and at birth, neonates are colonized by microbiota shaped by the same factors. Innovative approaches and methods are now needed to disentangle the complex interactions between lifetime exposures, the disruption of pregnancy, and colonization at birth. One such effort involved exposing fetal mice to prenatal stress, followed by C-section delivery and inoculation with distinct microbial communities [46]. In these studies, transplantation of microbiota from stressed dams into treatment naïve C-section delivered pups was sufficient to recapitulate phenotypes observed in stress-exposed offspring, establishing a mechanistic link between maternal microbiota and negative health outcomes in offspring [46].

A central argument in the debate regarding the safety and efficacy of vaginal seeding and fecal microbiota transplantation procedures hinges on whether birth-associated microbial exposure has any lasting health benefits, such that a lack of exposure prevents the initiation of a developmentally-important event [27, 28, 47, 48]. Recent strain-resolved microbiome analyses showed that maternal vaginal bacteria –*Lactobacillus crispatus*, *Atopobium vaginae*, *Gardnerella vaginalis*– are recovered from the stool of newborns, providing support for neonatal inheritance of maternal vaginal microbiota [49]. These bacteria are members of distinct community state types (CSTs) [50–52]. Specifically, CST I (L. crispatus) and CST IV (G. vaginalis, A. vaginae) exhibit distinct metabolic and immune properties in the female reproductive tract [53–56], suggesting that CST-specific properties could be transferred to offspring. We used our C-section and oral gavage protocol to inoculate mouse pups with either CST I or CST IV and assessed outcomes across development and into adulthood. Birth-associated exposure to CST I and CST IV resulted in transient colonization of the intestinal tract in C-section delivered mice and was sufficient to elicit CST-specific transcriptional signatures in the neonatal ileum, as well as sex-specific differences in adulthood [57]. Epidemiological studies demonstrated that maternal obesity and presence of vaginal *G. vaginalis* are two common risk factors associated with increased risk for adverse obstetric outcomes [58–63]. We established a two-hit model to determine whether maternal obesity and presence of vaginal *G. vaginalis*, alone or in combination, would affect the offspring postnatal response to CST I or CST IV exposure at birth [57]. Surprisingly, neonates that gestated in a two-hit environment showed a pathological immune response and decreased survival following exposure to CST IV, a pattern that was not observed in CST I exposed offspring. This pathological response to the postnatal microbiota was associated with disruption to the development of the placenta and fetal ileum in the two-hit neonates [57]. If these results bear any translational relevance, they underscore that prenatal exposures directly shape how offspring respond to their postnatal microbial environment and that one unforeseen consequence of high-risk pregnancies may be a pathological response to the colonizing microbiota and increased neonatal morbidity.

While this area of research will undoubtedly contribute significant insight into postnatal microbiota transplantation procedures and effects on health, this topic has also garnered a growing fascination in public discourse over the last several years. Reflecting a trickle-down effect on social attitudes, a recent study examining attitudes towards vaginal seeding reported that pregnant individuals viewed these procedures as replicating a natural process and may help reduce maternal shame and guilt about undergoing c-section [64]. This self-reported shame and guilt about undergoing a lifesaving procedure reflects a long cultural history of blaming mothers for negative outcomes in their children, particularly mothers from historically oppressed groups [65]. Moving forward, we must take great care in anticipating how research describing cesarean delivery as a ‘missed opportunity’ that can be ‘recovered’, ‘naturalized,’ ‘restored,’ and ‘reconstituted’ by vaginal seeding and fecal microbiota transplantation will be interpreted in popular discussions, and how the resultant discourse may inform policies on bodily autonomy and reproductive health in our current sociopolitical climate.

## Should we vaginally seed the neonatal microbiome after C-section?

### Maria Gloria Dominguez-Bello

C-section delivery impairs the intergenerational transfer of the maternal microbiome, altering natural colonization by pioneer communities of different infant epithelia in different body sites. C-section and pre or perinatal antibiotics are associated with increased risk of autoimmune and metabolic diseases in humans and in mice. Thus, as we perform medical practices that have collateral costs, we need to restore and minimize the damage.

I want to share three ideas regarding neonatal microbiota seeding at birth:

1. Nature design for fish, amphibians, reptiles, birds and mammals- entitles the offspring being born through a canal that is i) colonized by microbes and ii) shared with the defecation canal (cloaca) or adjacent to it (vagina). This non-random design favors transmission of maternal gut bacteria which will successfully colonize the offspring’s gut. Thus, it is not surprising that fecal transplant to newborns is effective in colonizing the infant gut [28].
2. In humans, the vaginal canal in the late third gestational trimester does contain bacteria shared with feces, and furthermore, shared also with oral, nose and skin sites [48]. This extraordinary opening of the vaginal ecosystem to colonization by bacteria from other sites is consistent with the provision of pioneer communities for all of the baby’s sites. Vaginal seeding of C-section babies normalizes the microbiome development in the infant gut, skin and mouth, making them resemble more those of vaginally born infants, than those of C-section born infants.
3. Given the serious consequences for the baby of impaired microbiota transmission at birth, restoration needs to be implemented with current state of the art, minimizing both infection risks for the baby and the costs of inaction. Why use “the real stuff”, and not wait until we understand better which are the exact bacteria and when to give them? Because of the complexity of biological fluids (we are still performing blood transfusions because we don’t know yet how to reconstitute blood from purified components).

Recapitulating natural exposures at birth after careful examination of the maternal vaginal health will not put the baby at higher risk of infections than vaginally born infants. In the crucial windows of infancy, correct programming will determine future health, and thus impacts and perturbations cannot be followed by inaction, but by rehabilitation to provide what was missing to restore organisms and ecosystem functions that are crucial for health.

## Should we restore the neonatal microbiome after C-section with fecal microbiota transplantation or probiotics?

### Katri Korpela

The gut microbiota is being increasingly recognized as an important part of human physiology and a significant contributor to health. This is especially true in infants, where the gut microbiota influences the developing immune system and overall physiology. Gut microbiota composition in infancy is associated with a range of health outcomes in later childhood, most prominently weight and immune health [66–72]. Factors known to disrupt normal gut microbiota development in infants – C-section birth, antibiotics, and lack of breastfeeding – are also associated with weight and immune development [14, 73–78]. The links between early-life antibiotic use with asthma and overweight have been shown to be mediated by gut microbiota [75, 79]. Furthermore, mouse experiments have shown that altering the gut microbiota at a young age alters metabolic and immunological development [80–83]. We have shown in humans that correcting aberrant gut microbiota development in infants reduces the risk of allergic disease in a high-allergy-risk cohort [84, 85], strongly indicating a causal role of gut microbiota in disease development. Overall, there is evidence that the gut microbiota plays a significant role in infant development, and promoting a healthy gut microbiota is warranted.

The case for microbiota restoration is especially clear for infants born by C-section. The important colonisers of infant gut and primary utilisers of breast milk oligosaccharides, *Bifidobacterium* and *Bacteroides* strains, are naturally transferred from the mother’s gut during birth [86]. They are non-spore-forming anaerobes, which have a poor ability to survive in the environment and are thus dependent on vertical transfer at birth. This transfer is disrupted by C-section birth [86], leading to reduced abundance and delayed colonisation of these important bacteria in the infant gut [87]. Alarmingly, intrapartum antibiotics administered to the mother have a similar impact on the infant gut microbiota as C-section birth [88, 89]. In addition, bifidobacteria are strongly negatively affected by antibiotics given to the infant [90]. Infants born by c-section, or exposed to intrapartum or postpartum antibiotics would very likely benefit from microbiota restoration.

Since the maternal gut is the natural source of microbes to the infant gut, maternal fecal microbiota transfer (FMT) is an effective method of microbiota restoration in CS-born neonates [28]. Mimicking the natural vertical transfer of microbiota at birth, FMT provides the infant with a large diversity of maternal microbes, and the ones adapted to the infant gut flourish and colonise essentially permanently [86]. On the contrary, inoculation of the infant with maternal vaginal microbes has limited efficacy: Two studies have shown that while swabbing the infant with vaginal microbes increases the relative abundance of lactobacilli, it fails to restore *Bacteroides* and other fecal bacteria [47, 48]. In a re-analysis of the data, we showed that the gut microbiota of vaginally seeded infants resembles that of untreated CS-born infants, rather than that of vaginally born infants [28]. Oral administration of maternal vaginal microbes to CS-born infants has been shown to produce no significant differences in gut microbiota compared to no treatment [91]. The vagina is a poor source of gut microbes [91, 92], likely due to the highly selective vaginal environment with a low pH that inhibits the growth of fecal microbes.

While maternal FMT is effective, it is unlikely to be a feasible standard solution for all neonates. Careful screening of the mother is required due to the potential of transmitting pathogens. Indeed, often the most common reason for intrapartum antibiotic treatment is maternal carriage of group B streptococcus, which can cause a dangerous infection in the infant. Infants of these mothers would benefit from microbiota restoration but FMT may not be a safe option. FMT from a universal donor could be a solution, and an intriguing question is whether FMT from a universal donor is as effective as maternal FMT. While there is some evidence to suggest that strains from an infant’s own mother may be particularly compatible with the infant’s gut [86], some mothers may harbor a suboptimal microbiota e.g. due to antibiotic use or disease. Further research is needed on FMT to infants.

Probiotics can be used to restore at least part of the normal infant gut microbiota [85, 93, 94]. Products containing infant gut-adapted bifidobacteria, such as *Bifidobacterium longum* subsp. *infantis*, *B. breve*, and *B. bifidum*, are likely to be useful. Since many such strains are currently on the market and safe, their use in C-section-born and antibiotic-exposed infants is highly recommendable. Short-term administration of probiotics to C-section-born neonates in the hospital appears promising [95] and warrants further study. Further development of probiotic products specifically for the neonate could provide a practical and cost-effective solution of microbiota restoration. However, it is possible that a small and standardized set of strains is not effective in all individuals regarding all health outcomes. It should be noted that restoration efforts are unlikely to be successful if the infant is not breastfed, as human milk oligosaccharides support the growth of the beneficial microbes.

The long-term health benefits of infant microbiota restoration should be investigated especially in different risk groups. Experimental restoration of gut microbiota provides a way to test causality in the link between infant gut microbiota and later health. Such long-term studies will take time, but there is sufficient evidence to adopt microbiota restoration practices before conclusive results on health benefits are available. In addition to microbiota restoration, however, efforts are needed to reduce microbiota disruption by promoting vaginal birth and breastfeeding, and carefully considering the need for routine prophylactic antibiotics during birth.

## Maternal Microbiome Transmission as Inspiration for Translational Research

### Trevor D. Lawley

Maternal transmission of microbes to their neonates is an unappreciated form of kinship involving microbes from gut, urogenital, oral and skin microbiotas [96]. Common maternally transmitted bacteria found in the gut microbiota of vaginally delivered babies who were breastfed include *Bacteroides* species, *Parabacteriodes* species, *Escherichia coli* and *Bifidobacteria* species that are highly adapted to co-colonize neonates as an ecosystem in symbiotic relationships that have co-evolved over many millennia [97]. After c-section birth, many of these bacterial species do not transmit to neonates, possibly due to the inhibitory effects of antibiotic exposure or competition from opportunistic environmental pathogens [12]. There is a need to develop a deep microbiological, evolutionary and ecological understanding of maternal transmission and its impact on microbiota acquisition and assembly in neonates to identify pioneering and keystone bacterial species and strains that provide beneficial properties.

The key challenge is to determine how these maternally transmitted microbes influence the longer-term growth, development and disease resistance of a baby [23]. Much of our current understanding of maternal transmission and early life microbiota assembly comes from large birth cohorts that track the microbiome of individual babies long-term while building a personalized biobank of biological samples linked to various metadata to enable large-scale, data-driven discovery [23, 98–101]. High-resolution metagenomics and anaerobic culturing coupled to whole-genome phylogenetic analysis are the most reliable approaches to identify maternal-neonate transmission events at the species and strain levels [12, 49, 102, 103]. However, the outputs of these studies are primarily associations that link bacterial taxa to clinical phenotypes, allowing for the generation of biological hypotheses, but the mechanisms remain poorly understood and unproven. There is a need to culture and biobank pioneering and keystone microbial species from neonates that potentially code for beneficial properties to enable experimental testing and biological discovery.

As an example of maternally transmitted bacteria, species of *Bifidobacterium* (phylum *Actinobacteria*) are highly adapted for maternal transmission and colonization of the neonatal intestine [104], through both faecal-oral and breastmilk-oral transmission [105]. There are many *Bifidobacterium* species described, however, *B. breve*, *B. bifidum* and *B. longum* are commonly found in the neonatal gut microbiotas in the UK [12], Sweden, Russia and USA [99]. Importantly, *B. longum* subsp. *infantis* is not common in neonates born in any of these Western world countries, but is the dominant founder bacterium in neonates from Low- and Middle-Income Countries Bangladesh [106], Gambia [107] and Malawi [108]. It is possible that *B. longum* subsp. *infantis* has been lost from Westernized human populations due to modern lifestyles, such as diets, antibiotics, etc. Bifidobacteria play a pioneering role in nucleating and shaping the gut microbiota assembly by supporting the acquisition and colonisation of new beneficial microbes through cooperation such as cross-feeding human milk oligosaccharides (HMOs) [109], or through the inhibition of pathogen colonisation [110]. More recently, *B. infantis* has been shown to harbour highly evolved pathways that metabolise breastmilk, releasing indole-3-lactic acid to shape immune development [111]. There are likely numerous more human-adapted functions and metabolites to be discovered from gut bacterial species beyond *Bifidobacteria* that have co-evolved to transmit from mother to child that could potentially be exploited therapeutically.

We need to innovate ways to protect and nurture a baby’s microbiota to optimize growth, development and disease resistance. Understanding the ecological processes and mechanisms of maternal transmission and early life microbiota acquisition and assembly holds the key to biological discovery to enable translational sciences. We need to consider alternatives to antibiotics that spare a baby’s microbiota, and also therapies that can recover/restore the microbiota after antibiotic therapy. Microbiota transplantation studies in neonates using undefined maternal microbiomes have been undertaken but longer-term we envision more sophisticated, defined approaches [48, 91]. A deep understanding of maternal transmission and neonate microbiome assembly will guide the discovery and development of a variety of novel products based on microbes or their metabolites, such as Live Bacterial Products, rationally designed prebiotics, small molecule bioactives and targeted phage therapies. Breastmilk is rich in immunoglobulins, lactoferrin, cytokines and growth factors and provides passive immunity to the infant while the immune system is developing [112]. Breastmilk is also enriched in Human Milk Oligosaccharides (HMOs) that serve as nutrients and energy for maternally transmitted bacteria, and potentially inducers of beneficial functions. We need to understand the interplay between neonatal gut microbiome assembly and breastmilk and will likely find bioactives derived from bacteria, potentially induced by breastmilk, with activities that act directly on human cells within the gut but also at systemic sites impacting immunological, metabolic and cognitive development. We believe maternal transmission is deeply evolved and there are opportunities to consider maternal transmission in a global context, including maternal microbiota transmission as evolutionarily conserved in humans but adapted to local lifestyles and cultures, to establish and strengthen Global Health and translational research on early life microbiotas.
